# Fingerprinting Evaluation and Gut Microbiota Regulation of Polysaccharides from Jujube (*Ziziphus jujuba* Mill.) Fruit

**DOI:** 10.3390/ijms24087239

**Published:** 2023-04-14

**Authors:** Zhenwei Li, Menglei Wu, Wenlong Wei, Yaling An, Yun Li, Qiuyi Wen, Daidi Zhang, Jianqing Zhang, Changliang Yao, Qirui Bi, De’an Guo

**Affiliations:** 1 Zhongshan Institute for Drug Discovery, Shanghai Institute of Materia Medica, Chinese Academy of Sciences, Zhongshan 528400, China; 2 School of Chinese Materia Medica, Nanjing University of Chinese Medicine, Nanjing 210023, China; 3 Shanghai Research Center for Modernization of Traditional Chinese Medicine, National Engineering Research Center of TCM Standardization Technology, Shanghai Institute of Materia Medica, Chinese Academy of Sciences, Shanghai 201203, China

**Keywords:** jujube fruit, polysaccharides, fingerprint, gut microbiota regulation

## Abstract

Jujube fruit was well-loved and praised by the broad masses due to its delicious taste, abundant nutritional value, and medicinal properties. Few studies reported the quality evaluation and gut microbiota regulation effect of polysaccharides of jujube fruits from different producing areas. In the present study, multi-level fingerprint profiling, including polysaccharides, oligosaccharides, and monosaccharides, was established for the quality evaluation of polysaccharides from jujube fruits. For polysaccharides, the total content in jujube fruits ranged from 1.31% to 2.22%, and the molecular weight distribution (MWD) ranged from 1.14 × 10^5^ to 1.73 × 10^6^ Da. The MWD fingerprint profiling of polysaccharides from eight producing areas was similar, but the profile of infrared spectroscopy (IR) showed differentiation. The characteristic signals were screened and used to establish a discrimination model for the identification of jujube fruits from different areas, and the accuracy of identification reached 100.00%. For oligosaccharides, the main components were galacturonic acid polymers (DP, 2–4), and the profile of oligosaccharides exhibited high similarity. The monosaccharides, GalA, Glc, and Ara, were the primary monosaccharides. Although the fingerprint of monosaccharides was semblable, the composing proportion of monosaccharides revealed significant differences. In addition, the polysaccharides of jujube fruits could regulate the gut microbiota composition and possess potential therapeutic effects on dysentery and nervous system diseases.

## 1. Introduction

Jujube (*Ziziphus jujuba* Mill.) belongs to the Rhamnaceae family, and its fruit is a popular food worldwide due to its abundant nutritional and health benefits [1]. Jujube is cultivated in tropical and subtropical regions, such as China, India, North Africa, and Middle Eastern countries. Forty species were discovered in the world, and the majority of species of jujube originated in China (mainly distributed in Henan, Hebei, Shandong, Shaanxi, Shanxi, Xinjiang, Ningxia, and Gansu Provinces) [2,3]. Studies demonstrated that jujube fruits had a variety of bioactive compounds such as phenolic acids, flavonoids, polysaccharides, mineral constituents, etc. [4,5]. In addition, they possessed antioxidant [6], anti-inflammatory [7], neuroprotective [8], anti-tumor [9], and immunomodulating [10] pharmacological activities. Polysaccharides are one of the crucial bioactive substances which account for about 7.9% of jujube fruit [11] and exhibit antioxidant activity [12], oral pathogenic bacteria-inhibiting [13], an anti-tumor effect [14], etc. However, few studies reported a satisfactory method that could be used for the quality evaluation of polysaccharides of jujube fruits from different producing areas. Furthermore, the studies on the regulatory function of gut microbiota of jujube fruit polysaccharides were limited. Therefore, the multi-level fingerprint profiling and gut microbiota regulation analysis of polysaccharides from jujube fruits are in urgent demand.

In order to explore the method for quality evaluation of polysaccharides from food/traditional Chinese medicines, innovative methods have been used for all-sided characterization through analyzing the profiling of crude, partially hydrolyzed, and completely hydrolyzed polysaccharides. For the analysis of crude polysaccharides, infrared spectroscopy was applied to evaluate the polysaccharides outline [15] and identify the functional groups [16]. Furthermore, HPGPC or HPSEC coupled with RID-MALLS was used for calculating the molecular weight distribution (MWD) of polysaccharides due to its splendid separating property and accuracy [17,18]. Infrared spectroscopy and RID-MALLS could generate the fingerprint of multiple batches of polysaccharides for consistency evaluation [19,20]. In order to further explore the composition information, the polysaccharides were partially hydrolyzed into oligosaccharides with a weak acid solution. Then hydrolysis solution was derivatized with ABEE for improving polarity, strengthening response intensity, and enhancing ultraviolet absorption of oligosaccharides [21]. The derivatized samples were analyzed by HILIC or UHPLC coupled with MS for depicting the oligosaccharides mapping and elucidating the structural information of oligosaccharides [22]. Monosaccharide composition was a crucial part of the structure identification and differentiation evaluation of polysaccharides. Polysaccharides were completely hydrolyzed with trifluoroacetic acid (weak destructivenessand volatility) to elucidate the monosaccharide composition. Monosaccharides could not be directly detected due to their strong polarity, poor absorption, and low ionization efficiency. PMP derivatization was beneficial for the detection of multifarious monosaccharides because of the advantages of derivative stability, moderate derivative condition, strong ultraviolet absorption, and easy ionization [23]. Usually, HPLC/UHPLC-UV or LC-MS/MS was used for qualitative and quantitative analysis of PMP derivatization monosaccharides [24,25].

Studies reported that food polysaccharides possess multiple biological benefits for physical health. It could regulate gut microbiota for maintaining intestinal homeostasis and treating an array of conditions [26,27]. Jujube polysaccharides could restore the gut microbiota profile of the colitis cancer mouse model induced by AOM/DSS and had the potential for the prevention and treatment of colorectal cancer [28]. Furthermore, jujube polysaccharides could regulate the composition of gut microbiota. It promoted *Megasphaera* and *unclassified_f_Veillonellaceae* and inhibited *Bacteroides*, *Lachnoclostridium*, *Parabacteroide,* etc. [29]. However, few studies compared the gut microbiota regulation effect of polysaccharides from different jujube fruits.

The jujube fruits from different producing areas (including Ningxia, (NX); Henan, (HN); Shaanxi, (SAX); Shandong, (SD); Xinjiang, (XJ); Hebei, (HB); Gansu, (GS) and Shanxi, (SX) Provinces) were collected. In this study, a multi-level evaluation strategy was developed for structural characterization and quality evaluation of polysaccharides from jujube fruits. The strategy was practiced at three tiers. For the polysaccharides analysis, infrared spectroscopy was used to identify characteristic absorption functional groups of polysaccharides, depict saccharide mapping and establish models on the basis of infrared signals for the identification of jujube fruits from different producing areas. Moreover, the MWD of polysaccharides was acquired by HPGPC-RID-MALLS, and fingerprint profiling was established and compared for difference analysis. For the oligosaccharides analysis, the oligosaccharide mapping was developed, and the oligosaccharide structures were characterized by UHPLC-Q-TOF/MS. For the monosaccharides analysis, the primary monosaccharides were quantified by UHPLC-UV. The multi-level profiles (polysaccharides mapping, oligosaccharides mapping, monosaccharides mapping) could demonstrate different insights to elucidate the characteristics of polysaccharides and screen splendid features for quality evaluation of polysaccharides. In addition, the gut microbiota regulation effect of different jujube fruits polysaccharides was evaluated and compared, and the ‘jujube fruits polysaccharides—gut microbiota—potential disease’ network was obtained. It is anticipated that the network could provide a reference for the development of healthy food and exploration of pharmacological activities of polysaccharides from jujube fruits.

## 2. Results and Discussion

### 2.1. Determination of Total Polysaccharides Content and MWD of Polysaccharides

#### 2.1.1. Comparison of Total Polysaccharides Content of Jujube Fruits

The content of total polysaccharides from jujube fruits was determined by the phonel–sulfate method. The methodology includes the estimation of inter-day and intra-day precision, stability, linearity, repeatability, and recovery. The results showed that the RSD % was lower than 2.2% and R = 0.9993, which indicated the methodology to be acceptable (Tables S1–S5, Figure S2). As exhibited in Figure S3, the total polysaccharides content in jujube fruits ranged from 1.31% to 2.22%. The content of total polysaccharides from NX was the highest, while the SX was the lowest. The content discrepancy existed in different areas; the order of average values was as the following: NX > HN > SAX > SD > XJ > HB > GS > SX > Others.

#### 2.1.2. Comparison of MWD of Polysaccharides from Jujube Fruits

**Figure 1:**
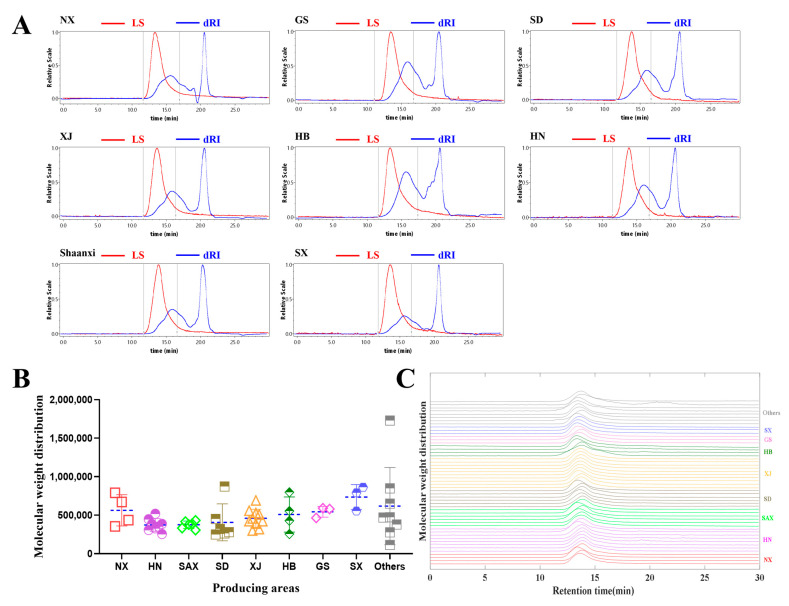
The MWD of polysaccharides of jujube fruits from different production bases by HPGPC-RID-MALLS. (**A**) Representative chromatogram of polysaccharides; (**B**) Distribution of MWD; (**C**) Fingerprint of polysaccharides.

**Figure 2:**
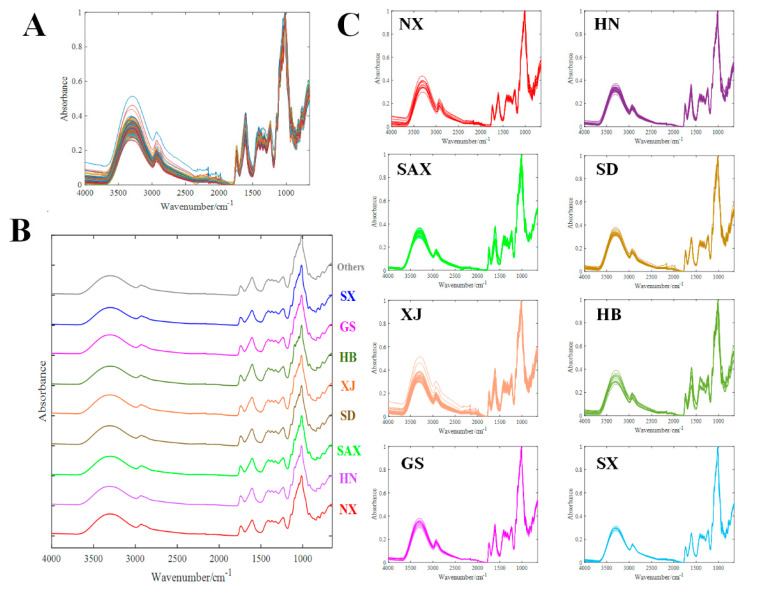
The spectra of polysaccharides of jujube fruits from different production bases by FT-IR. (**A**) Total fingerprint of polysaccharides; (**B**) Average map profiles of polysaccharides from different production bases; (**C**) Fingerprint of polysaccharides of jujube fruits from different production bases.

The MWDs of polysaccharides of jujube fruits from different producing areas were collected and calculated by HPGPC-RID-MALLS. The representative chromatograms of polysaccharides from jujube fruits were demonstrated, and one prime peak representing polysaccharides appeared between 12 min and 17 min (Figure 1A). The MWD of polysaccharides of jujube fruits ranged from 1.14 × 10^5^ to 1.73 × 10^6^ Da, and there were obvious differences among different production bases. The degree of polymerization of polysaccharides from SX was bigger than those of production areas (Figure 2B). The degree of polymerization of polysaccharides among different production areas exhibited differences. The fingerprint of MWD showed that the profile of polysaccharides from different producing areas was similar on the whole (Figure 2C), but there was still evident discrepancy existed in the same and different producing areas. The average value of MWD was in the following order: SX > NX > GS > HB > XJ > SD > SAX > HN.

### 2.2. Polysaccharides Profiling and Fingerprinting Evaluation by FT-IR

The spectra of polysaccharides from jujube fruits were collected in the range of 650 cm^−1^ to 1800 cm^−1^ to build the fingerprint for holistic evaluation by FT-IR. The total and different producing areas fingerprints of polysaccharides were exhibited in Figure 2A,B, and there were some spectral profile differences observed. The intraspecific difference between HB, XJ, and NX was larger than that of HN, SAX, SD, GS, and SX. Furthermore, the average map profiles of different producing areas were similar, and significant signal features were exhibited in the band between 650 cm^−1^ to 1800 cm^−1^, which allowed the identification of major chemical groups in polysaccharides (Figure 2C). The absorption band in the range of 3700 cm^−1^ to 3000 cm^−1^ was triggered by O-H stretching vibrations, and the absorption band at 2920–2930 cm^−1^ was caused by CH_2_-group stretching vibration. The carbonyl (C=O) and phenyl ring (C-C) triggered the stretching vibrations at 1743 cm^−1^ and 1605 cm^−1^, respectively [30]. A stretching band at 1000–1200 cm^−1^ showed the presence of C-O-C and C-O in carbohydrates. The relatively strong absorption at 1013 cm^−1^ also represented the featured absorption of polysaccharides, which showed the existence of the C–O group.

To distinguish the jujube fruits from different producing areas and evaluate the difference in polysaccharides, the unsupervised models and supervised models were established on the basis of characteristic spectral signals. Principal component analysis (PCA) as an unsupervised model was applied to display the distribution in PC1 and PC2 (Figure S4A). Whereafter, partial least squares-discriminant analysis (PLS-DA) was used to distinguish the jujube fruits from different producing areas, and a mass of differential signals (VIP > 1) was screened out to establish the discriminant model (Figure S4C). The established PLS-DA models passed the overfitting test (Figure S4B). After the confusion matrix analysis, the accuracy of identification achieved 100.00% for jujube fruits from different producing areas (Figure S4D).

### 2.3. Oligosaccharides Profiling Analysis

#### 2.3.1. Characterization of Oligosaccharides by High-Resolution Mass Spectrometry

**Figure 3:**
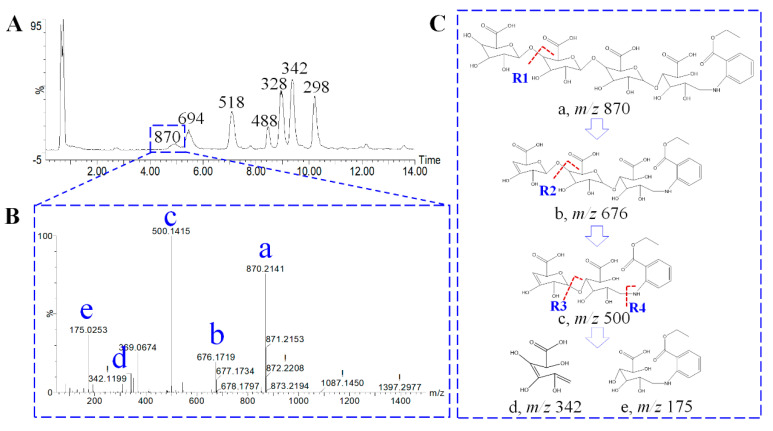
Characterization of oligosaccharides in the negative ion mode. (**A**) Base peak chromatogram of oligosaccharides; (**B**) MS/MS spectrum of *m/z* 870. (**C**) Speculative identification of characteristic fragment of *m/z* 870.

The partially hydrolyzed parameters, including trifluoroacetic acid concentration, hydrolysis temperature, and hydrolysis time, were optimized to acquire more kinds of oligosaccharides with high response intensity [31]. Then 1 mol/L trifluoroacetic acid, 100 °C hydrolysis temperature, and a 2 h hydrolysis time were used as the parameters for polysaccharides hydrolysis (Figure S5). After the derivatization with ABEE, the samples were injected into UHPLC-Q-TOF/MS. The base peak chromatogram of the oligosaccharides is exhibited in Figure 3A. Three monosaccharides (including arabinose, galacturonic acid, and glucose) and four oligosaccharides were characterized (Table 1). The oligosaccharides were mainly composed of galacturonic acid polymer (DP, 2–4). Take ABEE-four galacturonic acid polymers as an example; the characteristic fragments were expressly presented in MS/MS spectrum (Figure 3B). Tag a represented the [M-H]^−^ of ABEE-four galacturonic acid polymers, and the precursor ion was *m/z* 870.21. Four featured positions (R1, R2, R3, R4) were ruptured continuously, and the featured fragments such as *m/z* 676.17, *m/z* 500.14, *m/z* 342.11, *m/z* 175.02. were presented (Figure 3C).

#### 2.3.2. Distribution Profile and Fingerprint Evaluation of Oligosaccharides

**Figure 4:**
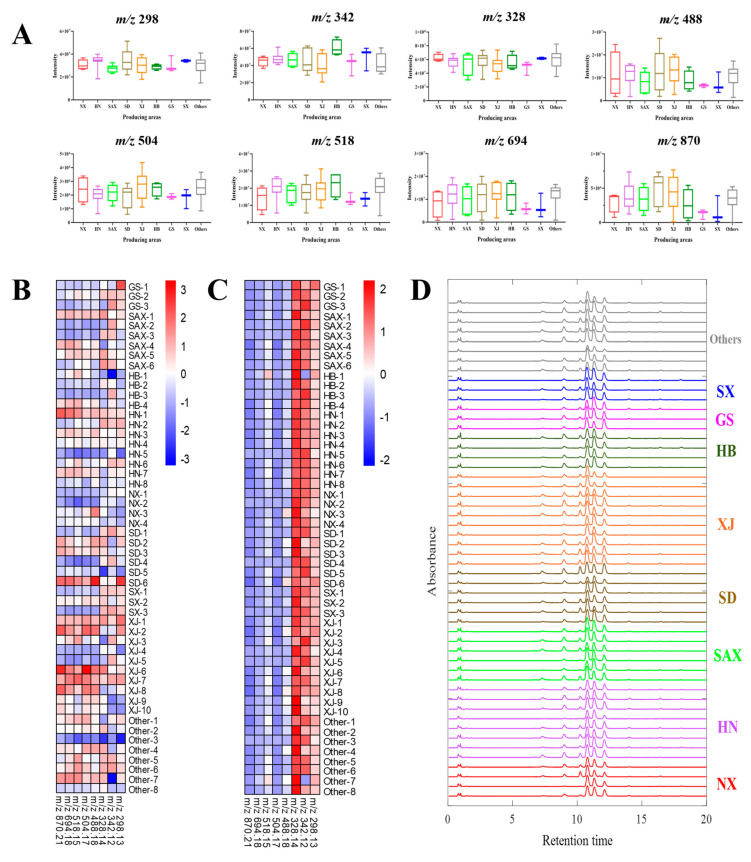
Distribution profile of oligosaccharides of jujube fruits from different producing areas. (**A**) Difference in response intensity of monosaccharides and oligosaccharides among different producing areas. (**B**) Longitudinal normalization of monosaccharides and oligosaccharides; (**C**) Horizontal normalization of monosaccharides and oligosaccharides; (**D**) Fingerprint profile of oligosaccharides in different producing areas.

According to the optimized conditions, the 52 batches of polysaccharides samples originating from eight producing areas were disposed of and injected into a high-resolution mass spectrometer. In order to compare the distribution difference of oligosaccharides in different producing areas, the box plots of each oligosaccharide were demonstrated (Figure 4A). Meanwhile, the heat maps (longitudinal normalization and horizontal normalization) are shown in Figure 4B,C. The results showed that the distribution profile of oligosaccharides among different producing areas was similar. The polysaccharides readily hydrolyzed to monosaccharides, resulting in a high abundance of arabinose, galacturonic acid, and glucose. The abundance of oligosaccharides from XJ, SD, HN, and SAX was stronger than those from SX, NX, GS, and HB. Subsequently, the fingerprint of oligosaccharides was drawn (Figure 4D), and the profile of oligosaccharides from eight producing areas was semblable.

### 2.4. Monosaccharides Profiling Analysis

**Figure 5:**
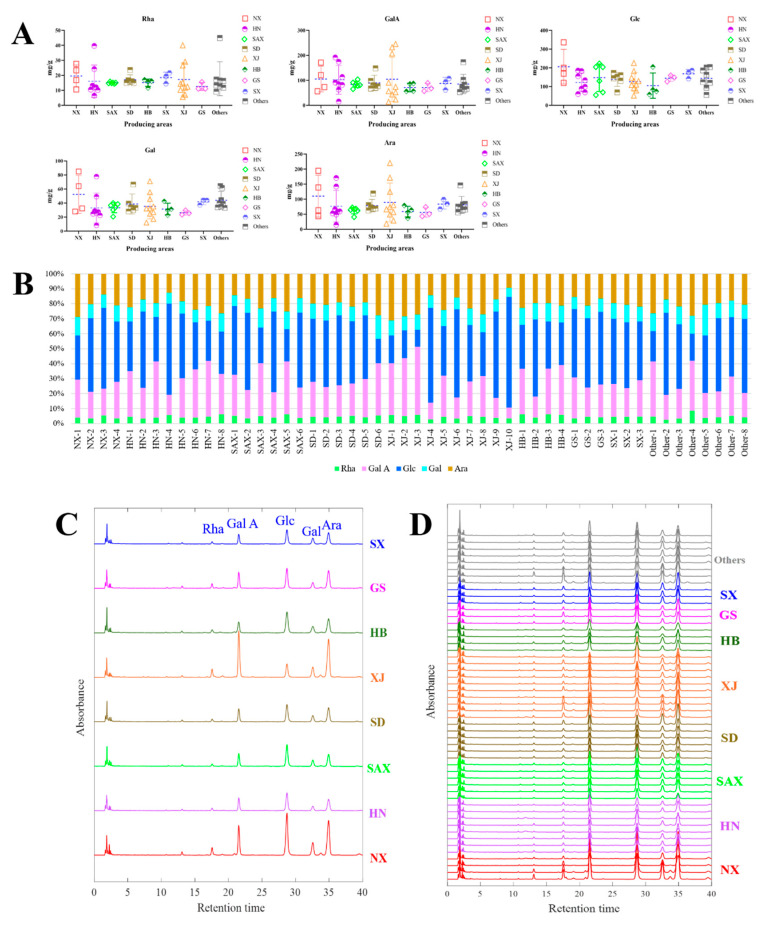
Monosaccharides profiling of jujube fruits from different producing areas. (**A**) Distribution of monosaccharides; (**B**) Distribution proportion of monosaccharides; (**C**) Representative chromatogram of monosaccharides; (**D**) Fingerprint of monosaccharides.

The polysaccharides were completely hydrolyzed and derivatized with PMP and then detected by UHPLC-UV. The methodology, including precision, repeatability, linearity, recovery, and stability, were examined, and the results were acceptable (Tables S6–S11, Figure S6). A total of 52 batch samples were detected, and the content of each monosaccharide was calculated and analyzed (Table S12). As shown in Figure 5A,B, the content of each monosaccharide distributed in different producing areas was similar, and the content of GalA, Glc, and Ara was higher than that of Rha and Gal. The content of Rha, GalA, Gal, and Ara showed significant intraspecific differences in XJ and HN. The composition proportion of monosaccharides presented remarkable differences. Furthermore, the representative chromatogram and fingerprint of monosaccharides were exhibited in Figure 5C,D. The results showed that the distribution profile of monosaccharides from different producing areas was approximately parallel. Glc, GalA, and Ara are the main peaks, and the absorption intensities of Rha and Gal are relatively low.

### 2.5. Modulatory Effects of Different Jujube Fruits Polysaccharides on Mice Gut Microbiota

**Figure 6:**
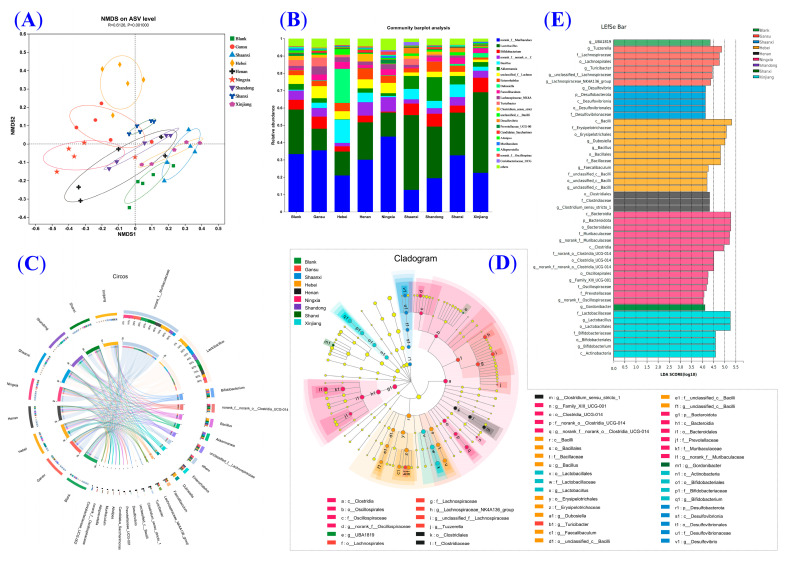
The modulatory effects of different jujube fruits polysaccharides on mice gut microbiota. (**A**) beta diversity of gut microbiota estimated by NMDS analysis on ASV level; (**B**,**C**) bar plot and Circos plot of microbiota community composition at genus level; (**D**,**E**) Cladogram plot and LDA score bar plot of LEfSe analysis of mice gut microbiota.

In an attempt to further explore and compare the modulatory effects of different jujube polysaccharides on gut microbiota, the mice feces were collected after 14 days of oral administration, followed by 16S rRNA sequencing. Table 2 demonstrates the alpha diversity of mice gut microbiota estimated by Chao, Shannon, Shannoneven, and pd indexes, which reflected the community richness, community diversity, community evenness, and phylogenetic diversity, respectively. As can be seen, most jujube fruit polysaccharides had no obvious effects on mice gut microbiota alpha diversity indexes, except for Shaanxi and Xinjiang, which exhibited a significantly decreased trend. For beta diversity (Figure 6A), the group of SAX and XJ were close to each other and closer to the blank control group than other groups, especially for samples from Hebei, Shanxi, and Gansu.

As revealed in Figure S7, the major phylum of mice gut microbiota were *Firmicutes*, *Bacteroidota*, *Actinobactenota*, and *Verrucomicrobiota*. Compared with phylum, the differences at the genus level among each jujube fruit polysaccharides group were much more significant (Figure 6B). The genus of *norank_f_Muribaculaceae* in SAX and SD decreased significantly, while *Lactobacillus* in SAX and XJ, *Bacillus* and *Dubosiella* in HB, *Enterorhabdus* in HN and SD, *Akkermansia* in SD showed an obvious increase compared with the blank control group. The relative abundance of each genus in different jujube fruits polysaccharides groups was visualized in Figure 6C by using a Circos plot. In a bid to further figure out the taxa that contribute to the differences among groups, linear discriminant analysis effect size (LEfSe) was conducted from phylum to genus level, with the LDA significant threshold of 4.0. As shown in Figure 6D, the number of taxa enriched in NX was the largest, followed by HB and XJ, while that in SD and SX were few. Specifically, the taxa enriched by jujube fruits polysaccharides included: from phylum *Bacteroidota* to genus *norank_f_Muribaculaceae* in NX, from phylum *Desulfobacterota* to genus *Desulfovibrio* in SX, from class *Actinobacteria* to genus *Bifidobacterium* in XJ, from class *Bacilli* to genus *Bacillus* in HB, and from order *Clostridiales* to genus *Clostridium_sensu_stricto_1* in HN.

### 2.6. Network Analysis of Jujube Fruits Polysaccharides-Gut Microbiota-Potential Diseases

**Figure 7:**
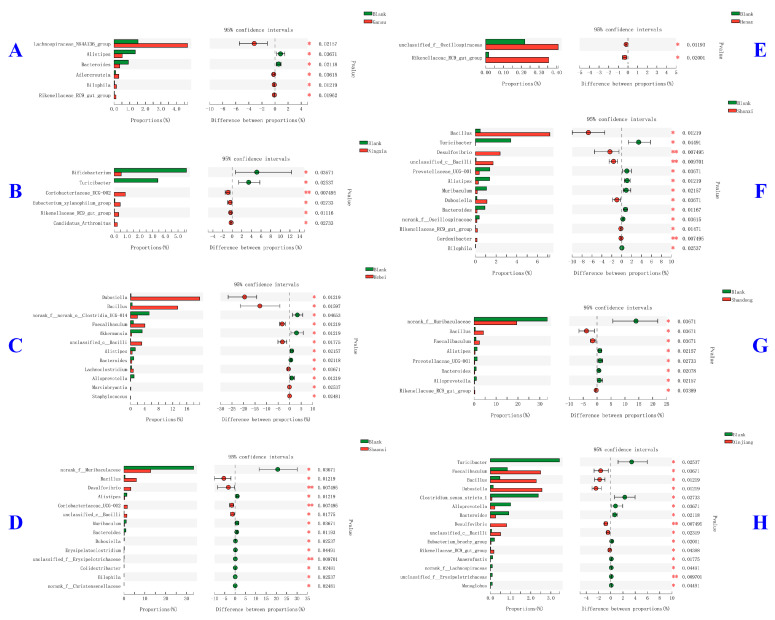
Comparative analysis of gut microbiota at genus level between the blank control group and each jujube fruit polysaccharides. (**A**) Gansu; (**B**) Ningxia; (**C**) Hebei; (**D**) Shaanxi; (**E**) Henan; (**F**) Shanxi; (**G**) Shandong; (**H**) Xinjiang. Significance level: * *p* < 0.05, ** *p* < 0.01.

It is well accepted that gut microbiota plays an important role in maintaining host health—the indigestible polysaccharides could act on gut microbiota and exert a beneficial effect. As shown in Figure 7, the effect of different jujube fruit polysaccharides on gut microbiota varied dramatically. The number of gut microbiotas with significant differences in jujube fruits polysaccharides extracted from HN, GS, and NX was relatively low, which indicated they possess a less significant effect on gut microbiota compared with SX, HB, SAX, and XJ. In addition, among the eight groups, HB jujube fruit polysaccharides showed the most obvious regulatory effect. The genus *Dubosiella* increased vastly from 0.14% in a blank control group to 19.67% in HB jujube fruit polysaccharides, and *Bacillus* increased from 0.47% to 13.38%. The genus *Dubosiella* has been reported to increase in the treatment of some diseases, such as sepsis-associated encephalopathy [32], type 2 diabetes mellitus [33], and rheumatoid arthritis [34]. In comparison, the genus *Bacillus* was considered one important kind of probiotic [35].

**Figure 8:**
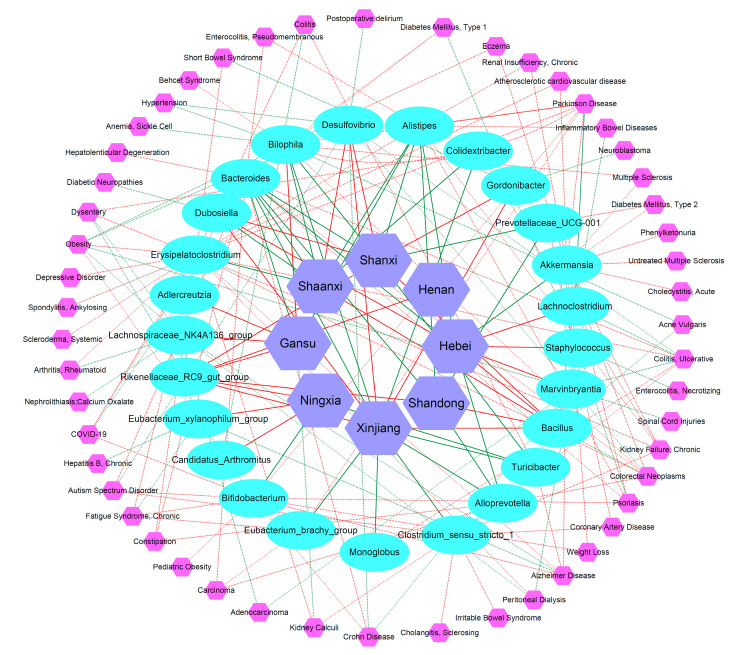
Network analysis of jujube fruit polysaccharides-gut microbiota-potential diseases.

Considering that one jujube fruit polysaccharide could act on multiple gut microbiotas, and one gut microbiota might change in abundance during the occurrence and development of various diseases, we further established a network to explore the relationship among jujube fruits polysaccharides, gut microbiotas, and potential diseases. The results are shown in Figure 8. For example, the relative abundance of *Rikenellaceae_RC9_gut_group* genus in mice gut after giving six of eight jujube fruits polysaccharides were all increased, including GS, NX, HN, SD, SX, and XJ. In addition, the genus *Rikenellaceae_RC9_gut_group* was reported to decrease in dysentery, indicating that jujube fruits polysaccharides might have potential therapeutic effects against dysentery by increasing the relative abundance of genus *Rikenellaceae_RC9_gut_group*. Other genera with a general upward trend included *Bacillus*, *Desulfovibrio,* and *Dubosiella*. While genera with a general downward trend composed of *Alistipes*, *Alloprevotella*, *Bacteroides,* and *Turicibacter*.

Moreover, jujube fruit polysaccharides could simultaneously upregulate or downregulate multiple gut microbiotas to exert a potential beneficial effect on a specific disease. For example, jujube fruit polysaccharides from HB could upregulate the abundance of *Lachnoclostridium* and *Marvinbryantia*, which both decreased in Parkinson’s disease; in the meantime, HB jujube fruit polysaccharides could also downregulate *Alistipes* and *Akkermansia*, which both increased in Parkinson’s disease. By regulating multiple gut microbiotas, jujube fruits polysaccharides mainly had potential therapeutic effects on the following diseases: Parkinson’s disease, Alzheimer’s disease, Autism spectrum disorder, ulcerative colitis, Crohn disease, colorectal neoplasms, chronic kidney failure, chronic fatigue syndrome, psoriasis, and COVID-19 (Figure S8). The beneficial effects might be related to the biological activities of jujube polysaccharides, including antioxidant, immunomodulatory, anti-tumor, and gastrointestinal-protective activity [36]. In addition, jujube fruit polysaccharides have been proven to have significant protective effects on chronic fatigue syndrome rat models [10] and chronic kidney disease rat models [37], anti-tumor effects on human colorectal carcinoma LoVo cells [14], and colorectal cancer mice models [28]. In the future, more in vitro and in vivo experiments are needed to verify these predicted results and explore the possible mechanisms of action from the perspectives of gut microbiota.

## 3. Materials and Methods

### 3.1. Materials

Acetonitrile was purchased from Merck KGaA (Merck, Darmstadt, Germany), and formic acid (FA) was purchased from Sigma-Aldrich (Sigma-Aldrich, St. Louis, MO, USA). Trifluoroacetic acid and 3-Methyl-1-phenyl-2-pyrazoline-5-one (PMP) were bought from Shanghai Chemical Reagent Co., LTD (Sinopharm, Shanghai, China). The deionized water (18.2 MΩ at 25 °C) was prepared by a Millipore Alpha-Q water purification system (Millipore, Bedford, MA, USA). Benzocaine was purchased from Sigma (Sigma-Aldrich, St. Louis, MO, USA). L-Rhamnose (Rha, No. WXBB6950V, purity ≥ 99.0%) and D-Galacturonic acid (GalA, No. BCBH7675V, purity ≥ 97.0%) were bought from Sigma (Sigma-Aldrich, St. Louis, MO, USA). D-Arabinose (Ara, No. 20170830, purity ≥ 98.0%) was purchased from Shanghai Chemical Reagent Co., LTD (Sinopharm, Shanghai, China). D-Glucose (Glc, No. 3696, purity ≥ 98.0%) and D-Galactose (Gal, No. 5460, purity ≥ 99.5%) were purchased from Shanghai Standard Technology Co., Ltd. (Nature Standard, Shanghai, China). The representative pictures of jujube fruits from different producing areas are displayed in Figure S1. The voucher specimens were deposited at the National Engineering Research Center of TCM Standardization Technology, Shanghai Institute of Materia Medica, Chinese Academy of Sciences, Shanghai, China.

### 3.2. Preparation of Total Polysaccharides, Hydrolyzed Oligosaccharides, and Monosaccharides

Polysaccharides: Jujube fruits were smashed and sieved with a No.3 griddle, then 5.0 g of powder was extracted with 75 mL of water by reflux extraction. The extraction temperature was 100 °C, and the extraction time was 4 h. The residue was refluxed again with 75 mL of water for 4 h, centrifuged, and the supernatant was pooled. Ethanol was added to make the ethanol concentration reach 85% for precipitating polysaccharides at 4 °C for 12 h. The deposit was redissolved with water and centrifuged. Solvent (chloroform:n-butyl alcohol = 5:1, *v*/*v*) was added into an aqueous solution of polysaccharides to remove protein three times. After the shock and centrifugation, the water layer was dried to acquire polysaccharide powders by freeze-drying [38] (Chang et al., 2022).

Oligosaccharides: Two milligrams of polysaccharide powder were weighed and placed into a penicillin bottle, 2 mL of TFA (1 mol/L) were added and then sealed for hydrolysis at 100 °C for 2 h. The TFA was removed by vacuum drying, and the residue was redissolved with 1 mL of water. Whereafter, 200 μL solution was fetched and mixed with 80 μL acetic acid, 80 μL 1.4 M sodium cyanoborohydride, and 400 μL 0.6 M ABEE for derivatization at 65 °C water bath for 2 h. The reaction was terminated by adding 500 μL water, and the reaction solution was extracted with diethyl ether for the removal of ABEE. The water layer was dried and redissolved with 500 μL 60% methanol for injection [39] (Wong et al., 2019).

Monosaccharides: Four milligrams of polysaccharide powder was accurately weighed and dissolved into 2 mL of water through ultrasonic (1130 W, 37 kHz) for 30 min. The solution was centrifuged at 14,000 rpm for 10 min, and 200 μL supernatant was hydrolyzed with 4 mol/L TFA at 110 °C for 4 h, of which 300 μL of the solution was dried to remove TFA. Then the residue was dissolved with 200 μL water, and then 100 μL NaOH (0.3 mol/L) and 100 μL 0.5 mol/L PMP-methanol were added for derivatization at 70 °C for 30 min. The reaction mixture was cooled, 100 μL HCl (0.3 mol/L) was added to adjust the pH, then 500 μL chloroform was added twice to remove PMP. After vortex oscillation and centrifugation, the upper layer was taken out for quantitative analysis.

### 3.3. Determination of Total Polysaccharides Content

The glucose standard was accurately weighed and dissolved with water to prepare the 90 μg/mL of the mother solution. Different volumes (0.2 mL, 0.4 mL, 0.6 mL, 0.8 mL, and 1 mL) were transferred into a 1 mL volumetric flask for constant volume. The different concentrations of solution were put into test tubes, then 1 mL of 5 % phenol-water solution and 5 mL of sulfuric acid were added to trigger the reaction under a water bath for 20 min. A blank control was prepared with 1 mL of water using the same method. The absorbance was determined under 488 nm by ultraviolet spectrophotometer for depicting the standard curve [40] (Yue et al., 2022).

In brief, 10 mg powder of polysaccharides was dissolved with 100 mL of water in a 40 °C water bath for 30 min. Then 1 mL of solution was taken out and disposed of with the above method. The absorption wavelength was set at 488 nm, and the total polysaccharides content was calculated on the basis of the standard curve.

### 3.4. Determination of MWD of Polysaccharides by HPGPC-RID-MALLS

An Agilent 1260 series HPLC system (Agilent Technologies, Palo Alto, CA, USA) combined with a refractive index detector (Wyatt, Optilab T-rEX) and a multiangle laser light scattering detector (MALLS, DAWN HELEOS-II;, Wyatt Technology Co., Santa Barbara, CA, USA) was used to measure the MWD of polysaccharides. PBS as the mobile phase was used to elute the polysaccharides with aTSK-gel GMPWxl (300 × 7.8 mm, 13 μm; TOSOH Corp., Tokyo, Japan) column. The injection volume was 50 μL, and the flow rate was set at 0.5 mL/min [41,42] (Yu et al., 2021; Liu et al., 2022). A total of 2 mg of polysaccharide powder was dissolved into 1 mL of PBS (25 mM NaH_2_PO_4_ + 25 mM Na_2_HPO_4_ + 100 mM NaCl) by ultrasonic dissolution for 15 min. Then the polysaccharides solution was centrifuged for injection.

### 3.5. Polysaccharides Profiling by FT-IR Spectroscopic Analysis

A Fourier transform infrared spectrometer equipped with a single bounce diamond crystal attenuated total reflectance accessory (ATR), deuterated triglycine sulfate (DTGS), and OMNIC 9.7.7 software was used for data acquisition. IR spectra were collected between 650 and 4000 cm^−1^ with a spectral resolution of 4 cm^−1^ and 32 scans using the ATR sampling platform. The room temperature was below 25 °C, and relative humidity was below 40%. Air was collected as background, and each sample was detected in triplicate to reduce errors under the same conditions.

### 3.6. Oligosaccharides Profiling Analysis

A Waters Xevo^®^ G2-S Q-TOF mass spectrometer (Waters, Manchester, UK) was connected to a UHPLC system via a Zpray^TM^ ESI source and equipped with an ACQUITY BEH C18 (2.1 × 100 mm, 1.8 μm) column maintained at 30 °C for separation. The mobile phase consisted of 0.1% FA-water (A) and 0.1% FA-acetonitrile (B) was used for elution according to the following gradient program: 0–5 min, 10–10 % (B); 5–18 min, 10–18% (B); 18–23 min, 18–25% (B); 28–35 min, 30–50% (B); 35–40 min, 50–90% (B). The flow rate was 0.3 mL/min, and the injection volume was 2 μL. The spectrum of oligosaccharides was gathered in negative ion mode, and the related parameters were listed as follows: capillary voltages, 2.5 kV; cone voltage, 40 V; cone gas flow, 30 L/h; source temperature, 150 °C; desolvation gas flow, 600 L/h. These data were acquired in the Fast DDA method in the range of 100–1500 Da in full scan with the scan time of 0.15 s and 50–1000 Da for MS/MS. Low-mass collision energy (LM CE) was set as 20–30 eV, and high-mass collision energy (HM CE) was set as 40–50 eV to obtain more characteristic fragmentations. MassLynx V4.1 software (Waters, Milford, MA, USA) was applied for data acquisition and processing.

### 3.7. Monosaccharides Profiling Analysis

An Agilent UHPLC 1290 (Agilent Technologies, USA) equipped with a DAD detector was applied for the quantification of monosaccharides. A Waters CORTECS C18^+^ (4.6 × 150 mm, 2.7 μm) column was applied for separation. The mobile phase comprised of 20 mM ammonium acetate-water (A) and acetonitrile (B) was applied for elution, and the gradient elution program was as follows: 0–30 min, 15–17% (B); 30–40 min, 17% (B). The column temperature was 25 °C, and the flow rate of 0.65 mL/min. The detection wavelength was 250 nm, and the injection volume was 2 μL.

### 3.8. Modulatory Effects of Jujube Fruits Polysaccharides on Mice Gut Microbiota

#### 3.8.1. The 16S rRNA Sequencing of Mice Feces

Six-week-old C57BL/6J mice with a body weight of 20 ± 2 g were obtained from Beijing Vital River Laboratory Animal Technology Co. Ltd. (Beijing, China). The mice were randomly divided into nine groups (*n* = 5) and orally given water or eight different jujube total polysaccharides with a dosage of 200 mg/kg for 14 days. Then the mice stool samples were collected and stored at −80 °C for 16S rRNA sequencing. The DNA extraction, v3–v4 region amplification, and sequencing were conducted according to the manufacturer’s instructions at Majorbio Bio-Pharm Technology Co. Ltd. (Shanghai, China). The 16S rRNA sequencing data was analyzed using the Qiime2 [43] (Bolyen et al., 2019) pipeline with recommended parameters on the online cloud platform of Majorbio (Shanghai, China).

#### 3.8.2. Network Analysis of Jujube Fruits Polysaccharides-Gut Microbiota-Potential Diseases

After processing these 16S rRNA sequencing data, the Wilcoxon rank-sum test between the blank control group and each jujube fruit polysaccharides group was conducted for comparative analysis at the genus level. A genus with *p* < 0.05 was considered as significantly different and searched in the gutMDisorder v2.0 online database for gut microbiota associated-diseases [44] (Qi et al., 2022). The diseases belonging to the search type of “gut microbiota associated with phenotype” with the alteration trend opposite to the polysaccharides-gut microbiota were filtered. Then the network information of jujube fruit polysaccharides-gut microbiota-potential diseases was generated and visualized by the software of Cytoscape.

## 4. Conclusions

The multi-dimensional evaluation strategy of polysaccharides was developed for the quality evaluation of jujube fruits from different producing areas. The fingerprints of polysaccharides (IR/GPC-MALLs), oligosaccharides (LC-MS), and monosaccharides (UHPLC-UV) were established to evaluate the difference of jujube fruits polysaccharides from eight producing areas. Although the profile of fingerprints in different levels was similar, the characteristic signal intensity, polymerization degree, oligosaccharides abundance, and compositional proportion of monosaccharides exhibited visible differences. According to the monosaccharide proportions, oligosaccharide abundance, and MWD, we suspect that Glc, GalA, and Ara were the main monosaccharides, and the galacturonic acid polymer was the primary substance in polysaccharides. Furthermore, a discrimination model was established based on the characteristic signals of IR, and the jujube fruits from different producing areas could be well characterized. In addition, jujube fruit polysaccharides could improve the homeostasis of the bacterial community and play a potential role in the treatment of deficiency of Qi (vital energy), blood diseases, and nervous system diseases. Future work could focus on the discovery and separation of active polysaccharides, which have important practical significance for the drug development of polysaccharides.

## Figures and Tables

**Table 1:** Identification of oligosaccharides from partially hydrolyzed polysaccharides.

RT	*m/z*	Formula	Addition	Composition	DP
10.21	298.13	C_14_H_20_NO_6_	M-H	Arabinose	1
9.36	342.12	C_15_H_20_NO_8_	M-H	Galacturonic acid	1
8.92	328.14	C_15_H_22_NO_7_	M-H	Glucose	1
8.46	488.17	C_21_H_30_NO_12_	M-H	Two glucoses	2
7.08	518.15	C_21_H_28_NO_14_	M-H	Two galacturonic acids	2
5.46	694.18	C_27_H_36_NO_20_	M-H	Three galacturonic acids	3
4.97	870.21	C_33_H_44_NO_26_	M-H	Four galacturonic acids	4

**Table 2:** Alpha diversity of the bacterial community of mice feces after being given different jujube fruits polysaccharides.

Group	Chao	Shannon	Shannoneven	pd
Blank	177.7 ± 24.5	3.707 ± 0.372	0.717 ± 0.062	16.99 ± 1.63
Gansu	169.2 ± 14.0	3.782 ± 0.220	0.739 ± 0.045	16.51 ± 1.03
Hebei	169.8 ± 25.0	3.599 ± 0.192	0.704 ± 0.031	16.87 ± 1.98
Henan	186.4 ± 27.1	3.812 ± 0.414	0.732 ± 0.077	16.96 ± 1.56
Ningxia	178.3 ± 19.7	4.129 ± 0.278	0.797 ± 0.040	16.79 ± 1.02
Shaanxi	131.4 ± 25.3 *	2.665 ± 0.640 *	0.548 ± 0.108 *	13.92 ± 1.85 *
Shandong	163.6 ± 22.5	3.23 ± 0.428	0.635 ± 0.074	16.11 ± 1.54
Shanxi	162.8 ± 15.4	3.479 ± 0.275	0.686 ± 0.049	16.77 ± 0.91
Xinjiang	127 ± 14.1 *	2.843 ± 0.797	0.586 ± 0.155	13.71 ± 1.06 *

*: *p* < 0.05 compared with a blank group.

## Data Availability

Data available on request from the authors.

## Supplementary Materials

The following supporting information can be downloaded at: www.mdpi.com/article/10.3390/ijms24087239/s1.
