# Supplementary material for: Fingerprinting Evaluation and Gut Microbiota Regulation of Polysaccharides from Jujube (Ziziphus jujuba Mill.) Fruit
Source: Int J Mol Sci. 2023 Apr 14;24(8):7239. doi: 10.3390/ijms24087239 (PMC10138826; doi:10.3390/ijms24087239)
Supplement: Supplementary file 1 [file ijms-24-07239-s001.zip › ijms-2328634-supplementary.pdf]

Fingerprinting evaluation and gut microbiota regulation of polysaccharides from jujube (*Ziziphus jujuba* Mill.) fruit

Zhenwei Li [2]†, Wenlong Wei [3]†, Menglei Wu [1,3]†, Yaling An [3], Yun Li [3], Qiuyi Wen [2], Daidi Zhang [2], Jianqing Zhang [3], Changliang Yao [3], Qirui Bi [3], De-an Guo [1,2,3]*

Affiliation

[1] School of Chinese Materia Medica, Nanjing University of Chinese Medicine, Nanjing, Jiang Su 210023, China

[2] Zhongshan Institute for Drug Discovery, Shanghai Institute of Materia Medica, Chinese Academy of Sciences, Zhongshan 528400, China

[3] Shanghai Research Center for Modernization of Traditional Chinese Medicine, National Engineering Research Center of TCM Standardization Technology, Shanghai Institute of Materia Medica, Chinese Academy of Sciences, Shanghai 201203, China

† These authors contributed equally to this work

Correspondence:

Prof. De-an Guo, Shanghai Research Center for Modernization of Traditional Chinese Medicine, National Engineering Research Center of TCM Standardization Technology, Shanghai Institute of Materia Medica, Chinese Academy of Sciences, Haike Road 501, Shanghai 201203, China.

Email: daguo@simm.ac.cn Phone: +86 21 50271516 Fax: +86 21 50271516

Table S1 The result of intra-day precision

Content of total polysaccharide（%）							
1	2	3	4	5	6	Mean	RSD%
76.097	74.021	77.266	72.971	76.450	76.590	75.566	2.22

Table S2 The result of repeatability

Content of total polysaccharide（%）							
1	2	3	4	5	6	Mean	RSD%
76.097	74.881	74.436	76.104	76.837	78.646	76.167	1.97

Table S3 The result of stability

Content of total polysaccharide（%）								
0min	10min	30min	60min	90min	120min	240min	Mean	RSD%
76.104	75.966	75.787	75.652	75.372	75.029	74.700	75.516	0.68

Table S4 The result of sampling recovery rate

Content of total polysaccharide（%）							
1	2	3	4	5	6	Mean	RSD%
100.118	101.864	99.372	100.801	97.051	98.724	99.655	1.69

Table S5 The result of linearity

Regression equation	R2	R
y = 6.1826x + 0.071	0.9985	0.9993

Table S6 The result of inter-day precision

	1	2	3	Mean	RSD%
Rha	4.657	4.559	4.633	4.616	1.11
Gal A	21.096	21.754	23.151	22.000	4.77
Glc	12.867	12.779	13.036	12.894	1.01
Gal	8.125	8.098	8.154	8.126	0.34
Ara	21.212	21.968	21.947	21.709	1.98

Table S7 The result of intra-day precision

	1	2	3	4	5	6	Mean	RSD%
Rha	150.75	152.784	154.023	155.111	156.168	155.607	154.074	1.32
Gal A	896.812	895.766	891.594	888.485	884.652	880.393	889.617	0.72
Glc	476.502	475.458	477.728	484.052	487.982	487.858	481.597	1.19
Gal	558.064	559.774	561.928	563.258	564.389	565.955	562.228	0.52
Ara	1754.657	1758.679	1763.159	1766.631	1767.952	1770.888	1763.661	0.35

Table S8 The result of repeatability

	Content of monosaccharide（mg/g）							RSD%
	1	2	3	4	5	6	Mean	
Rha	3.935	4.129	4.041	4.029	4.007	3.887	4.005	2.12
Gal A	24.619	25.662	24.840	24.658	24.443	23.356	24.597	3.02
Glc	12.881	13.097	13.066	13.081	12.906	12.676	12.951	1.27
Gal	7.041	7.214	7.159	7.197	7.114	6.969	7.116	1.34
Ara	21.865	22.664	22.215	22.245	22.013	21.263	22.044	2.12

Table S9 The result of stability

	Peak area of monosaccharide（mAU）										RSD%
	0h	2h	4h	6h	8h	10h	12h	18h	24h	Mean	
Rha	134.247	134.518	135.83	136.81	136.383	137.275	137.469	128.344	131.115	134.666	2.29
Gal A	1441.76	1433.978	1429.645	1422.48	1412.575	1406.264	1399.575	1369.096	1357.653	1408.114	2.05
Glc	391.156	398.258	395.162	399.186	396.643	398.87	388.099	396.239	400.452	396.007	1.02
Gal	457.235	457.389	458.615	457.924	457.208	459.413	458.807	457.044	456.227	457.762	0.22
Ara	1493.807	1493.501	1494.458	1496.173	1491.968	1492.193	1492.706	1488.824	1490.134	1492.640	0.15

Table S10 The result of recovery

	Content of monosaccharide（mg）							RSD%
	1	2	3	4	5	6	Mean	
Rha	83.603	103.755	116.273	79.964	112.225	116.536	102.06	16.08
Gal A	86.074	101.192	114.024	75.710	107.193	104.398	98.10	0.15
Glc	92.479	95.931	106.859	89.645	106.299	105.415	99.44	7.72
Gal	95.025	97.446	104.469	95.126	106.259	105.358	100.61	5.27
Ara	89.583	89.583	89.583	89.583	89.583	89.583	100.12	9.60

Table S11 The result of linearity

	Regression equation	R2	R
Rha	y = 8899.9x - 7.7208	0.9982	0.9991
Gal A	y = 12149x - 52.927	0.9994	0.9997
Glc	y = 8165.8x - 12.468	0.9997	0.9999
Gal	y = 13846x - 4.3903	0.9996	0.9997
Ara	y = 16351x + 1.0085	0.9991	0.9996

Table S12 The content of monosaccharides by UHPLC-UV

	Rha	Gal A	Glc	Gal	Ara
	(mg/g)				
NX-1	27.68	170.98	199.97	85.04	194.52
NX-2	23.32	120.91	336.08	64.10	138.92
NX-3	16.80	56.43	169.07	28.07	43.73
NX-4	10.63	72.84	120.05	32.42	63.01
HN-1	13.14	91.69	99.26	28.68	66.33
HN-2	10.20	63.21	155.62	24.60	52.80
HN-3	12.07	113.93	89.46	27.47	59.72
HN-4	6.47	15.52	69.46	8.25	14.62
HN-5	12.04	82.02	134.60	25.52	57.23
HN-6	24.22	191.80	185.88	49.24	142.48
HN-7	10.54	84.72	60.61	22.59	48.96
HN-8	39.67	174.57	183.17	77.78	171.00
SAX-1	14.43	78.68	130.30	20.63	40.76
SAX-2	15.12	81.11	222.94	40.04	72.14
SAX-3	14.75	103.50	69.63	36.73	68.31
SAX-4	15.08	65.06	204.35	33.68	62.28
SAX-5	15.80	90.81	54.79	30.32	64.39
SAX-6	14.84	82.79	204.12	38.59	66.43
SD-1	14.89	80.38	143.81	34.10	68.08
SD-2	16.76	76.99	173.04	39.56	80.49
SD-3	15.03	68.71	152.06	28.20	62.66
SD-4	15.93	67.49	130.37	30.06	69.12
SD-5	16.11	97.81	161.11	33.39	72.89
SD-6	23.62	148.36	69.34	66.52	118.95
XJ-1	40.05	245.97	129.51	71.16	220.44
XJ-2	29.10	232.61	110.92	55.38	171.51
XJ-3	26.60	207.90	52.16	45.74	125.00
XJ-4	5.60	23.35	129.85	17.31	29.30
XJ-5	15.15	90.91	109.43	35.13	80.31
XJ-6	10.34	43.85	183.13	24.83	49.44
XJ-7	11.82	57.06	91.93	26.89	57.08

XJ-8	12.53	74.32	80.16	32.33	74.27
XJ-9	14.34	52.39	226.15	32.13	66.29
XJ-10	7.08	14.42	147.62	12.09	18.55
HB-1	17.03	85.56	83.07	31.26	64.00
HB-2	16.14	56.07	205.14	42.57	78.82
HB-3	11.59	56.83	57.76	22.61	36.64
HB-4	15.73	87.58	74.36	29.82	56.44
GS-1	11.14	89.13	148.11	25.46	50.98
GS-2	15.52	68.12	159.98	29.68	73.84
GS-3	11.06	57.04	126.75	23.34	43.16
SX-1	19.36	94.21	186.24	44.12	84.45
SX-2	14.41	61.38	141.77	37.21	67.23
SX-3	21.63	107.70	176.03	44.17	98.99
Other-1	13.37	101.99	55.93	33.21	72.92
Other-2	9.15	62.40	204.46	33.77	64.37
Other-3	12.62	74.24	163.01	44.53	82.50
Other-4	44.94	172.93	93.90	61.74	146.08
Other-5	11.76	53.11	120.57	64.65	65.14
Other-6	16.58	66.07	186.62	39.02	75.41
Other-7	16.36	82.95	123.92	34.92	56.36
Other-8	17.28	66.50	202.06	39.88	84.35

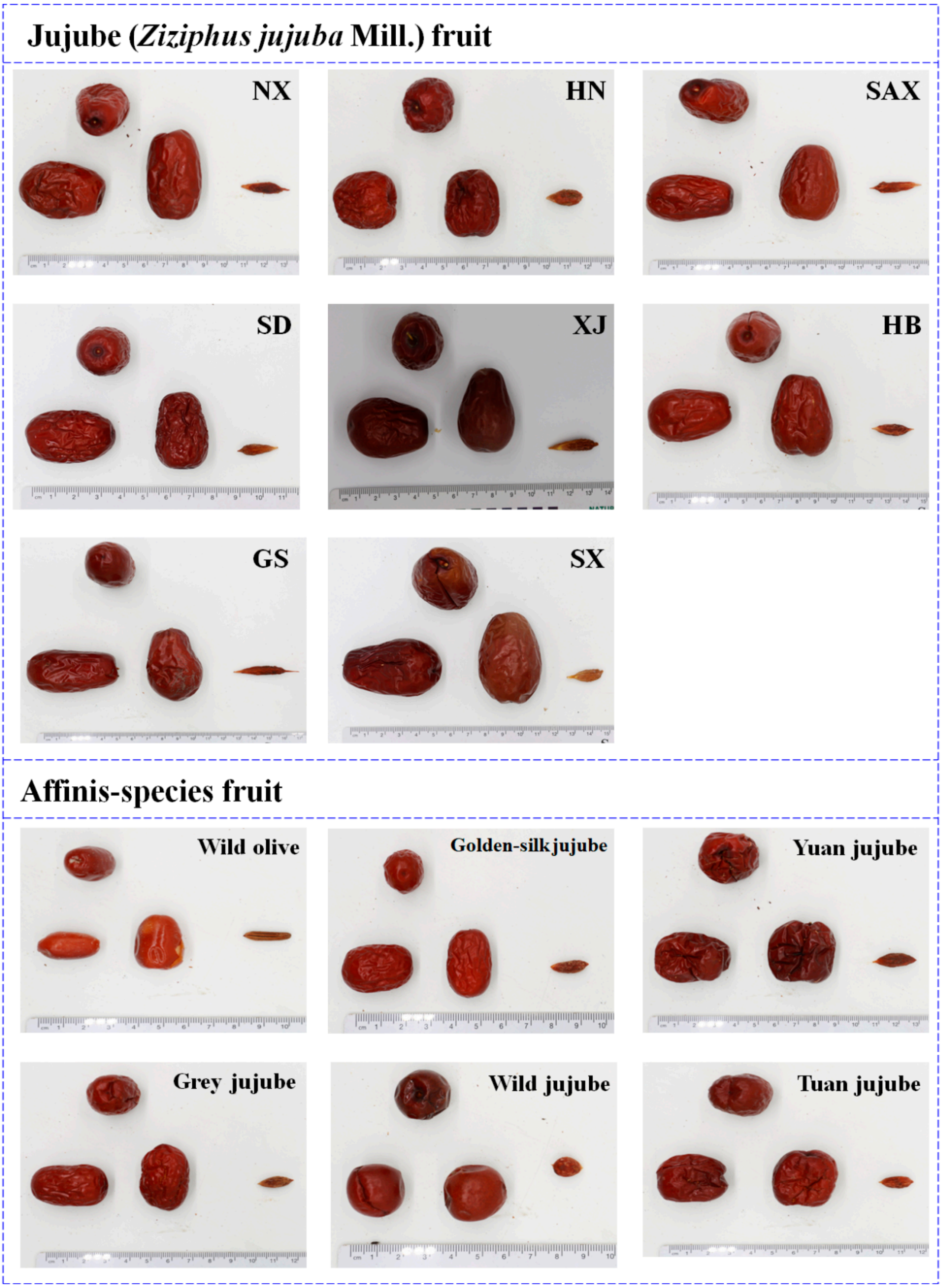

Figure S1. The representative pictures of jujube fruits from different producing areas and affinis species fruits (Ningxia, (NX); Henan, (HN); Shaanxi, (SAX); Shandong, (SD); Xinjiang, (XJ); Hebei, (HB); Gansu, (GS) and Shanxi, (SX) Province)

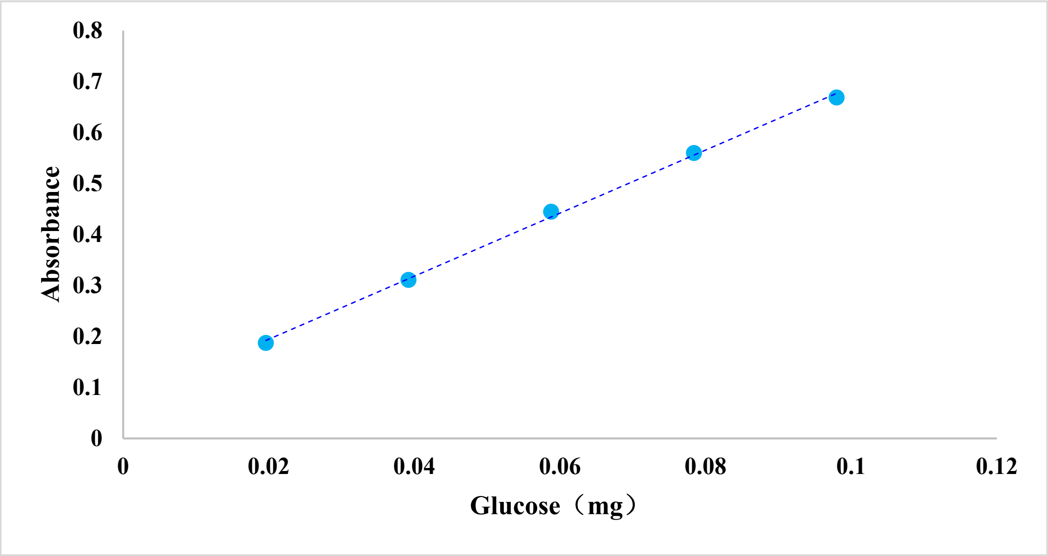

Figure S2. The linear equation of glucose

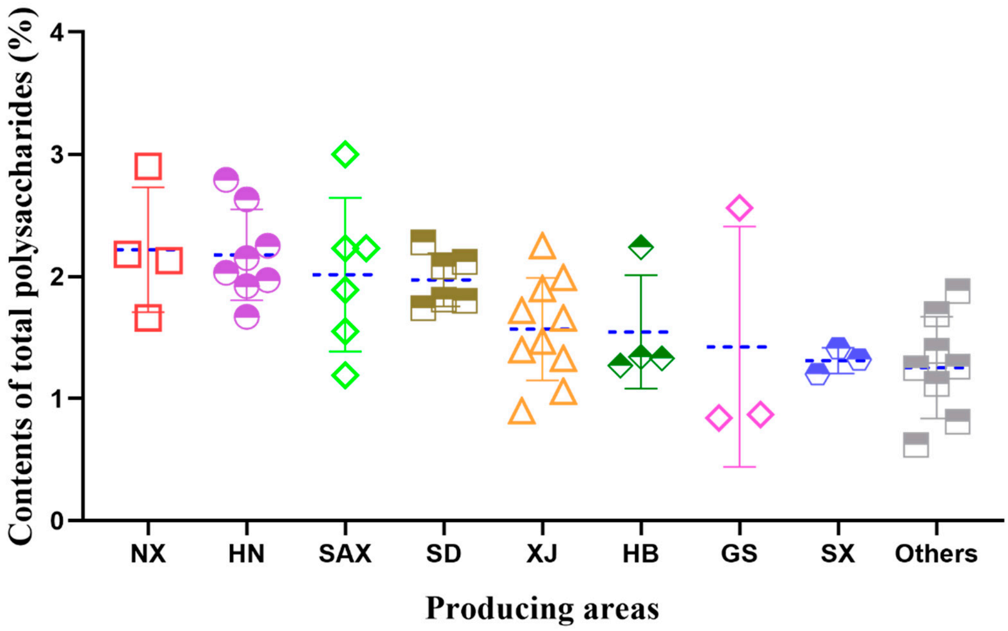

Figure S3. The content of total polysaccharides from jujube fruits

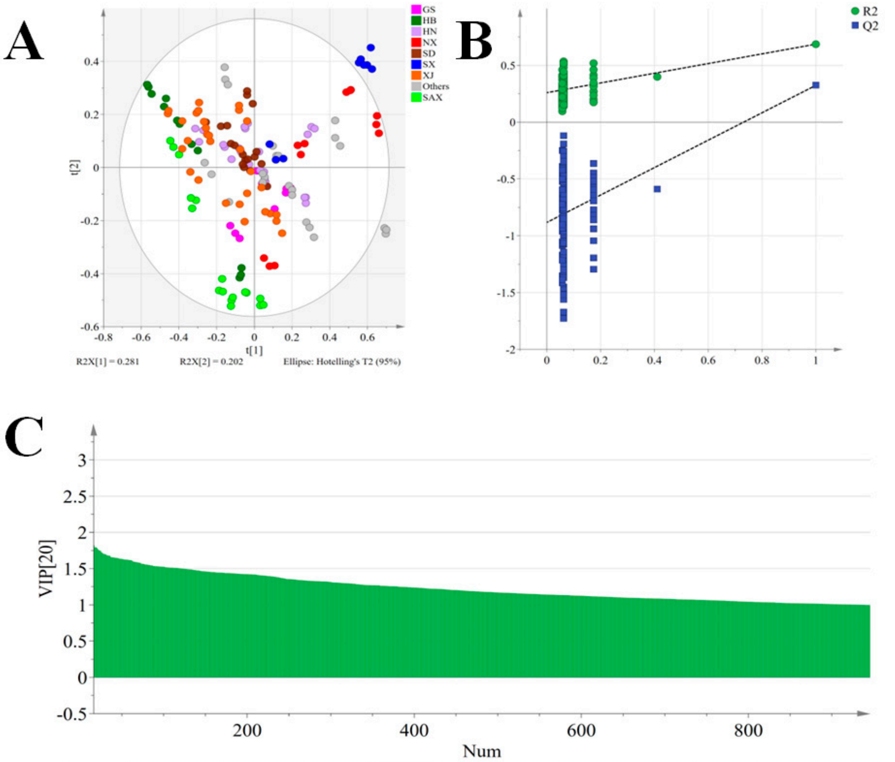

D

	Members	Correct	NX	HN	SAX	SD	XJ	HB	GS	SX	Others
**NX**	12	100%	12	0	0	0	0	0	0	0	0
**HN**	24	100%	0	24	0	0	0	0	0	0	0
**SAX**	18	100%	0	0	18	0	0	0	0	0	0
**SD**	18	100%	0	0	0	18	0	0	0	0	0
**XJ**	30	100%	0	0	0	0	30	0	0	0	0
**HB**	12	100%	0	0	0	0	0	12	0	0	0
**GS**	9	100%	0	0	0	0	0	0	9	0	0
**SX**	9	100%	0	0	0	0	0	0	0	9	0
**Others**	24	100%	0	0	0	0	0	0	0	0	24
Total	156	**100%**	12	24	18	18	30	12	9	9	24

Figure S4. The PCA and PLS-DA analysis of polysaccharides from jujube fruits. (A) PCA analysis; (B) Permutations of PLS-DA; (C) VIP ≥ 1; (D) Confusion matrix analysis for identification of jujube fruits from eight producing areas.

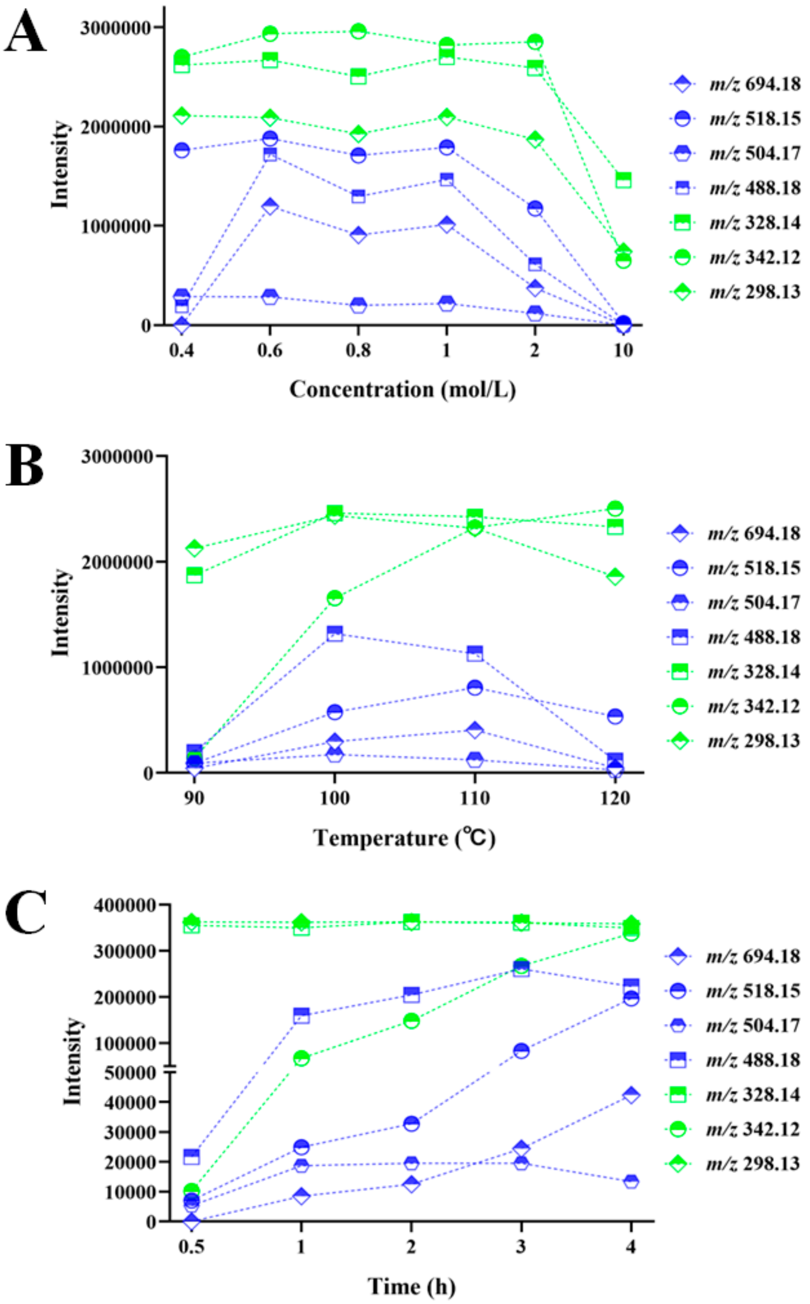

Figure S5. The optimization of partially hydrolyzed condition of polysaccharides

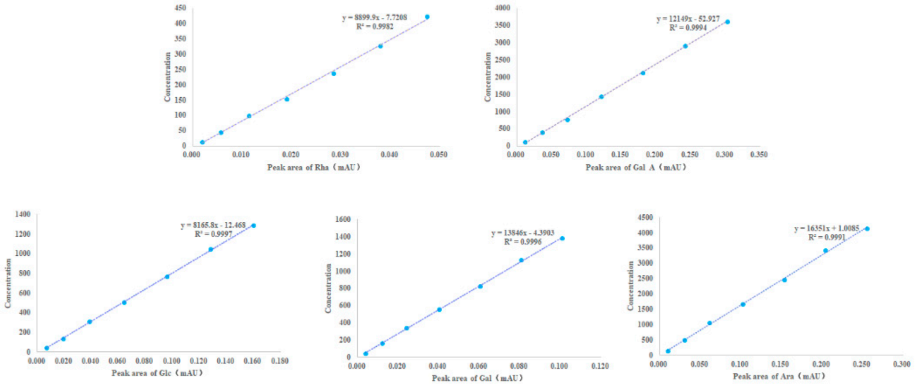

Figure S6. The linear equation of monosaccharides

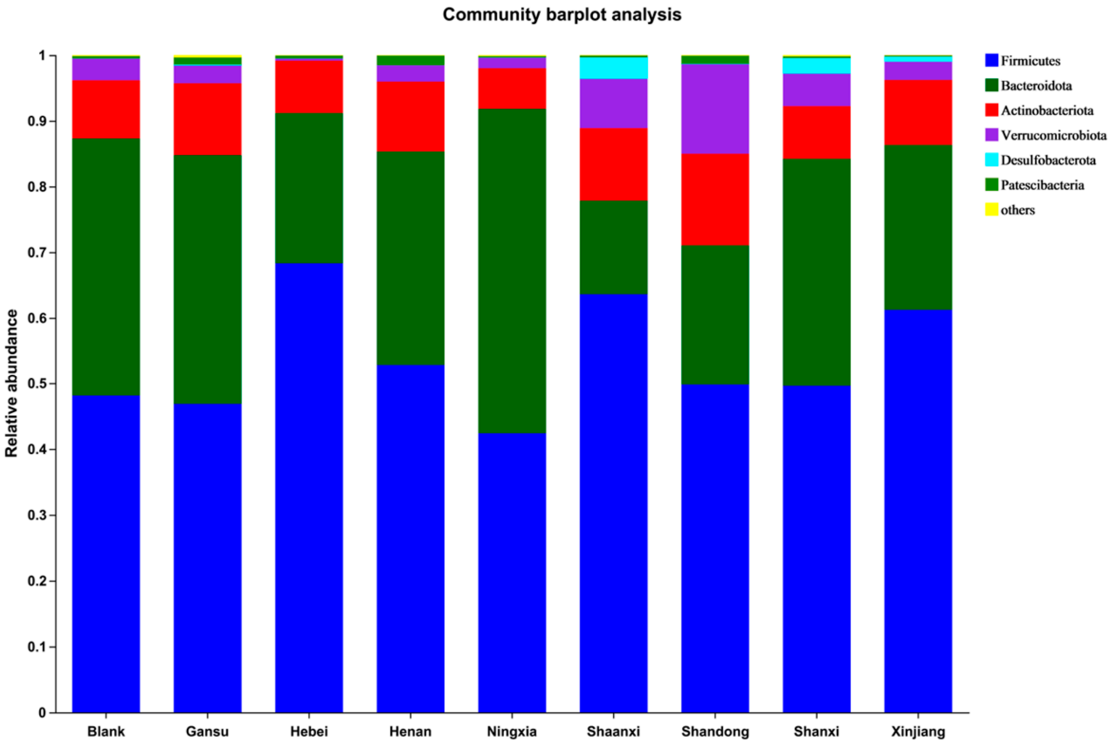

Figure S7. Bar plot of microbiota community composition at phylum level

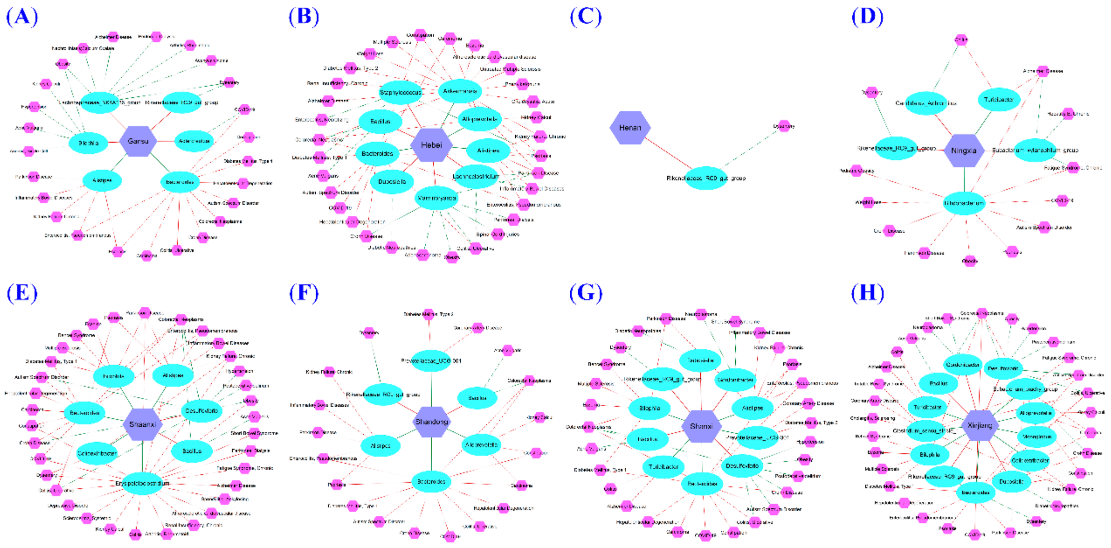

Figure S8. Network analysis of 'polysaccharides-gut microbiota-potential diseases' of jujube fruits from different producing areas
